# Dexmedetomidine in combination with morphine improves postoperative analgesia and sleep quality in elderly patients after open abdominal surgery: A pilot randomized control trial

**DOI:** 10.1371/journal.pone.0202008

**Published:** 2018-08-14

**Authors:** Huai-Jin Li, Chun-Jing Li, Xiao-Na Wei, Jian Hu, Dong-Liang Mu, Dong-Xin Wang

**Affiliations:** 1 Department of Anesthesiology and Critical Care, Peking University First Hospital, Beijing, China; 2 Department of Anesthesiology, Handan Central Hospital, Handan, Hebei, China; London Health Sciences Centre, CANADA

## Abstract

**Background:**

Dexmedetomidine in combination with opioids has been used for postoperative analgesia. The purpose of this study was to investigate the impacts of dexmedetomidine supplemented intravenous analgesia on morphine consumption and subjective sleep quality in elderly patients after open abdominal surgery.

**Methods:**

This was a pilot randomized controlled trial. 58 elderly patients (age ≥ 60 years) who underwent open abdominal surgery were randomized to receive either dexmedetomidine supplemented morphine analgesia (0.5 mg/ml morphine plus 2 μg/ml dexmedetomidine in 100 ml normal saline, DEX group) or morphine analgesia (0.5 mg/ml morphine in 100 ml normal saline, CTRL group) for 72 hours after surgery. Patient-controlled analgesia pump was programmed to deliver a 2ml bolus with a lockout interval of 8 minutes and a background infusion at a rate of 1 ml/h. The primary endpoint was 72-hour morphine consumption. Secondary endpoints included pain intensity, subjective sleep quality, and 30-day complications and mortality after surgery.

**Results:**

The 72-hour morphine consumption was lower in the DEX group than in the CTRL group (median 39.0 mg [interquartile range 37.3, 41.0] in the DEX group vs. 49.0 mg [45.5, 50.0] in the CTRL group; median difference -9.0 mg [95% CI -10.0, -6.0], P < 0.001). The intensity of pain within 48 hours was lower (P<0.001 at 4, 12 and 48 hours, P = 0.007 at 24 hours) whereas the subjective quality of sleep was higher (P = 0.031 during the night of surgery and P<0.001 during the 1^st^ night after surgery, respectively) in the DEX group than in the CTRL group. The incidence of 30-day complications did not differ significantly between groups, but it was slightly lower in the DEX group (P = 0.060). There were no significant differences between groups regarding 30-day mortality and the incidences of adverse events.

**Conclusions:**

For elderly patients after open abdominal surgery, dexmedetomidine supplemented analgesia decreases morphine consumption, improves analgesic effects and subjective sleep quality without increasing adverse events.

**Trial registration:**

Chinese Clinical Trial Registry ChiCTR-IPR-14005620.

## Introduction

Despite many efforts to improve analgesia, 45% to 86% of patients report moderate to severe pain after surgery [1–2]. Opioids are the mainstay of perioperative analgesia; however, up to 80% of patients suffer from adverse effects [1–3]. Multimodal analgesia is recommended to reduce opioid consumption and related adverse events [4]. Non-opioid analgesics, such as non-steroidal anti-inflammatory drugs (NSAIDs), ketamine and nefopam, are used to achieve these goals [5–8]. However, contraindications and safety concerns limit their widespread use. For example, cyclooxygenase-2 inhibitors are not approved for patients with ischemic cardio-cerebral vascular disease because they may increase the risk of cardiovascular events [5–6]; the use of ketamine is associated with higher risk of neurological and psychological events [7–8]; nefopam is not recommend for the elderly because its anticholinergic properties made it “notoriously deliriogenic” [8]. Therefore, use of these drugs are limited in the elderly who have higher prevalace of cerebral and cardiovascular disaese and are more prone to develop postoperative neuropsychological events [5–10].

As an alternative, dexmedetomidine (a highly selective α-2 receptor agonist) shows some advantages in pain management. Studies found that combined use of dexmedetomidine for acute pain after surgery reduces opioid consumption and improves analgesic effects [11–18]. However, care must be taken when explaining these results. Firstly, most of the studies were performed in young patients. Data in elderly patients are insufficient [13–18]. Secondly, safety outcomes were not reported in detail in the majority of available studies [13–16]. In patients after lung surgery, dexmedetomidine infusion produces sedation and lowers heart rate and systolic blood pressure, possibly due to a high dose [11]. In healthy volunteers, sedative dose dexmedetomidine inhibits the ventilatory response to hypoxia and hypercapnia [19]. Therefore, safety remains a great concern in using dexmedetomidine for analgesia. On the other hand, low-dose dexmedetomidine infusion improves sleep quality in elderly patients admitted to the intensive care unit (ICU) after surgery [20]. Dexmedetomidine for postoperative analgesia might also improve patient’s sleep quality, but evidences in this aspect are still lacking.

The purpose of this pilot study was to investigate the effects of dexmeditomidine in combination with morphine on analgesia, sleep quality and safety outcomes in elderly patients after open abdominal surgery.

## Materials and methods

This randomized, double-blinded, placebo-controlled pilot trial with two parallel arms was designed to investigate the superiority of combined dexmedetomidine-morphine analgesia. The study protocol was approved by the Clinical Research Ethics Committee of Peking University First Hospital (2014–024) and registered with Chinese Clinical Trial Registry (http://www.chictr.org.cn, identifier ChiCTR-IPR-14005620). Written informed consent was obtained from all enrolled patients. The study was conducted in the Peking University First Hospital (Beijing, China).

### Patient recruitment

Potential participants were screened the day before surgery. The inclusion criteria were patients of 60 years or older who were scheduled to undergo elective open abdominal surgery. Patients who met any of the following criteria were excluded: (1) bodyweight less than 50 kg or more than 90 kg; (2) history of schizophrenia; (3) presence of sick sinus syndrome, severe bradycardia (heart rate < 50 beats per minute), or atrioventricular block before surgery; (4) preoperative chronic renal failure (requirement of renal replacement therapy); (5) preoperative severe hepatic disease (Child-Pugh class C); and (6) predicted duration of surgery < 2 hours.

### Randomization and blinding

Random numbers were generated using software (SAS 9.2, SAS Institute Inc, Cary, NC) in a 1:1 ratio. These numbers were then sealed in sequentially numbered envelopes and stored at the site of investigation until the end of the study. During the study period, consecutively recruited patients were randomly divided into two groups by a study coordinator who prepared the study drugs according to randomization results in the envelopes before surgery. The study coordinator did not participate in anesthesia, perioperative care and postoperative follow-up of the enrolled patients. All study drugs were contained in the same 100 ml colorless reservoir bags and administered by the same brand analgesia devices (GEMSTAR®; Hospira, Inc., Lake Forest, IL, USA).

All patients, healthcare team members and investigators were blinded to study group assignment. In case of emergency (such as rapid deterioration of patients’ condition), investigators or care-givers could ask unmasking of group allocation or stop study drug administration. These conditions would be recorded and patients were included in the intention-to-treat analysis.

### Anesthesia and postoperative analgesia

Total intravenous anesthesia was provided for all patients. Anesthesia was induced and maintained with target-controlled infusion of remifentanil and propofol. Rocuronium or cisatracurium was administered to maintain muscle relaxation. Bispectral index was maintained between 40 and 60 during surgery. Blood pressure was maintained within 20% from baseline. Morphine 0.1 mg/kg was administered 0.5 hour before the end of surgery in order to control pain and relieve remifentanil-induced hyperalgesia. Patients were monitored in the post-anesthesia care unit for at least 30 minutes before being sent back to the general wards; otherwise, they were admitted to the intensive care unit after surgery.

Patient-controlled intravenous analgesia (PCIA) was provided for all patients from end of anesthesia until 72 hours after surgery. For patients in the control (CTRL) group, PCIA was established with 0.5 mg/ml morphine in 100 ml normal saline; while for those in the dexmedetomidine (DEX) group, it was established with 0.5 mg/ml morphine plus 2 μg/ml dexmedetomidine in 100 ml normal saline. For all patients, PCIA was programmed to deliver a 2ml bolus with a lockout interval of 8 minutes and a background infusion at a rate of 1 ml/h.

The target of postoperative analgesia was to maintain a Numeric Rating Scale (NRS, an 11-point scale where 0 indicated no pain and 10 the worst possible pain) [13] pain score at rest ≤ 3. For patients with NRS pain score > 3, PCIA bolus was firstly administered; in case of no significant improvement after three consecutive boluses, extra morphine 2–4 mg per time (maximum 0.1 mg/kg per hour) was administered and repeated. For patient in the general ward and without contraindications, flurbiprofen axetil 50–100 mg could also be given intravenously in case of NRS pain score > 3.

### Outcomes

The primary endpoint was the 72-hour morphine consumption, which was defined as cumulative morphine consumption (including PCIA dose and additional dose) from end of anesthesia until 72 hours after surgery. Secondary endpoints included NRS pain score, cumulative morphine consumption, NRS score of subjective sleep quality, and use of flurbiprofen axetil at various time-points after surgery. Other outcomes included the occurrence of complications within 30 postoperative days, the percentage of intensive care unit admission, the length of stay in hospital after surgery, and 30-day mortality.

Cumulative morphine consumption and NRS pain score (at rest and with movement) were assessed at 4, 12, 24, 48, and 72 hours after surgery [14]. NRS score of subjective sleep quality was assessed during the first 3 postoperative days. Patients were asked to compare their current sleep quality with their sleep at home and then ranked their current sleep quality according to a numeric rating scale (NRS, a 11-point scale where 0 indicates the worst sleep and 10 the best sleep) [20–21]. Delirium was assessed daily with the Confusion Assessment Method for the Intensive Care Unit during the first five postoperative days [20–22]. Patients were then followed up weekly (by telephone interview after hospital discharge) until 30 days after surgery. Postoperative complications were generally defined as newly occurred medical events that had unfavorable effects on patients’ recovery and required therapeutic intervention. All complications were diagnosed by attending intensivists or surgeons [23–25].

Adverse events were monitored from end of anesthesia until 72 hours after surgery, i.e., during the period of study drug administration. Tachycardia was defined as heart rate > 100 beats per minute (bpm) or a 30% increase from baseline. Bradycardia was defined as heart rate < 50 bpm or a 30% decrease from baseline. Hypertension was defined as systolic blood pressure > 180 mmHg or a 30% increase from baseline. Hypotension was defined as systolic blood pressure < 90 mmHg or 30% decrease from baseline. Over sedation was defined as Richmond Agitation Sedation Scale (RASS) ≤ -2[26]. Emergence agitation was defined as RASS ≥ +2 within 30 minutes after extubation [27]. Respiratory depression was defined as respiratory rate < 8 bpm or SpO_2_ < 92% [12]. Postoperative nausea and vomiting (PONV) indicated the occurrence of nausea, retching or vomiting.

### Statistical analysis

#### Sample size calculation

In a previous study, a 24-hour infusion of dexmedetomidine (at a rate of 0.08 μg/kg/h) decreased morphine consumption by 30% [14]. Considering that our patients were old and PCIA was used for 3 days, the dexmedetomidine concentration in the PCIA formula was decreased and, therefore, the background infusion rate was lower (a background infusion rate of 2 μg/h, that was about 0.02 to 0.04 μg/kg/h depending on patient’s body weight from 50 to 90 kg) in the present study than in previous ones [9,14,16–18]. We assumed that the addition of dexmedetomidine would reduce morphine consumption by 20%, i.e., from 50±10 mg in the CTRL group to 40±10 mg in the DEX group. With the significance set at 0.05 and power set at 80%, the sample size required to detect the difference was 24 patients in each arm. Taking into account a dropout rate of about 15%, we planned to enroll 28 patients in each arm.

#### Outcome analysis

Numeric variables (cumulative morphine consumption, NRS pain score, and NRS score of subjective sleep quality) were compared with independent sample t-test or Mann-Whitney U-test. Categorical variables (incidence of postoperative complications and adverse events) were compared with Chi-square test or Fisher’s exact test. The median differences (and 95% confidence intervals for the differences) were calculated with the Hodges-Lehmann estimator. Time-to-event variables (length of stay in ICU or hospital) were analyzed with Kaplan-Meier method and compared by log-rank test. All tests were two tailed and P values less than 0.05 were statistically significant. Bonferroni method was used to control type I error for multiple testing data. Statistical analyses were performed with the SPSS 14.0 software (SPSS, Inc., Chicago, IL) and SAS 9.2 software (SAS Institute, Cary, NC).

## Results

**Fig 1:**
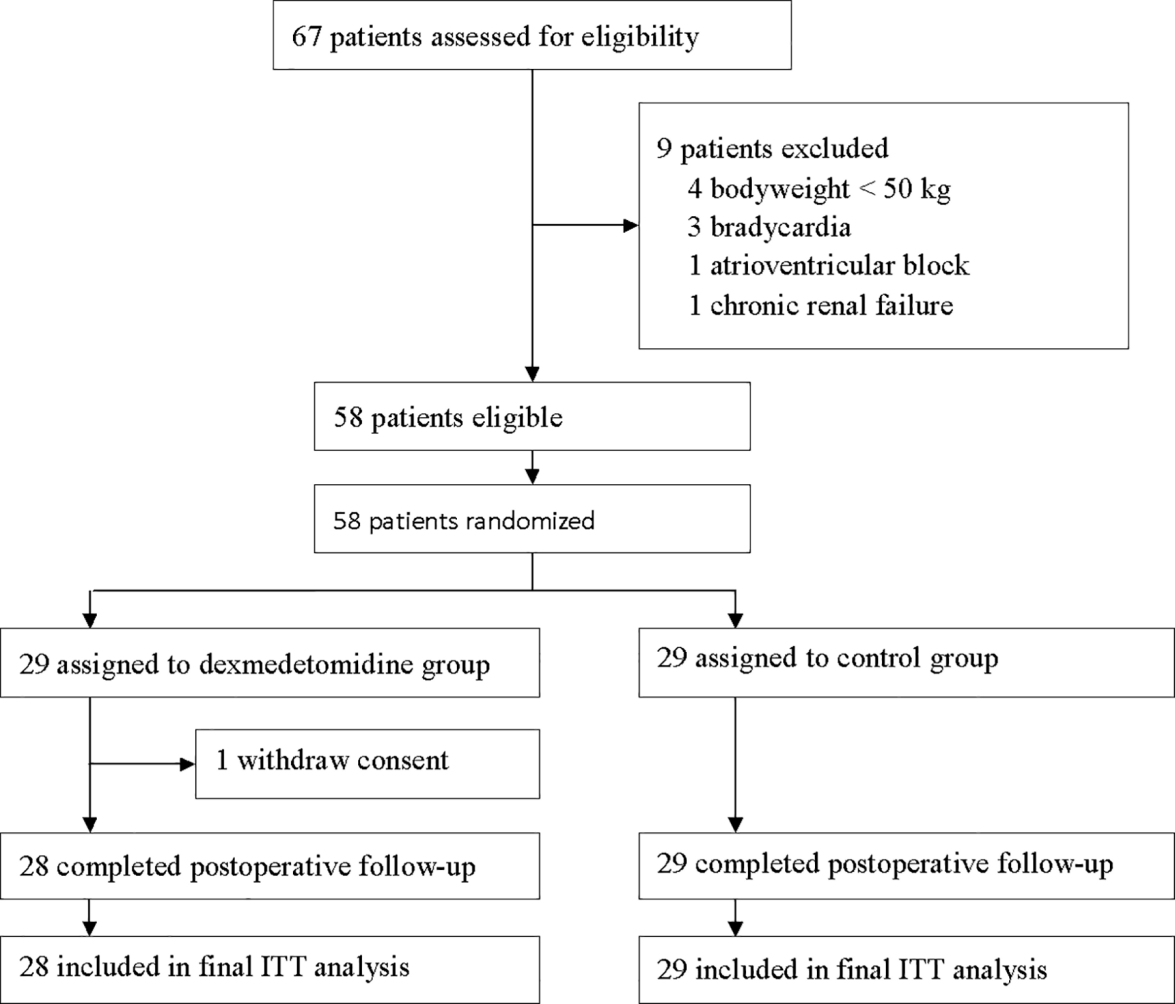
Flowchart of the study. ITT = intention-to-treat.

From December 15, 2014 to September 15, 2015, 82 patients were screened; of these, 58 were enrolled and randomized (Fig 1). In the DEX group, one patient withdrew consent and refused postoperative follow-up. Other patients completed study and were included in the final analysis. Baseline characteristics and intraoperative variables were well matched between the two groups (Tables 1 and 2).

**Table 1:** Baseline characteristics.

	CTRL Group (N = 29)	DEX Group (N = 28)
Age (year)	67.2 ± 5.7	69.2 ± 7.4
Gender (female)	14.0 (48.3%)	11.0 (39.3%)
BMI (kg/m^2^)	23.5 ± 0.5	24.1 ± 0.5
Preoperative history		
Stroke	2 (6.9%)	1 (3.4%)
Transient ischemic attack	0 (0.0%)	1 (3.6%)
Coronary artery disease	2 (6.9%)	3 (10.7%)
Hypertension	6 (20.7%)	9 (32.1%)
Hypertrophic cardiomyopathy	1 (3.4%)	0 (0.0%)
Diabetes	2 (6.9%)	1 (3.6%)
Asthma	1 (3.4%)	1 (3.6%)
Chronic smoking ^a^	2 (6.9%)	6 (21.4%)
Alcoholism ^b^	5 (17.2%)	4 (14.3%)
Charlson comorbidity index	4.3 ± 0.2	4.7 ± 0.2
Preoperative ASA classification		
I	4 (13.8%)	3 (10.7%)
II	24 (82.8%)	23 (82.1%)
III	1 (3.4%)	2 (7.1%)
Preoperative NYHA Classification		
I	25 (86.2%)	26 (92.9%)
II	4 (13.8%)	2 (7.1%)
Surgery for malignant tumor	26 (89.7%)	26 (92.9%)

Results are presented as mean ± SD or number (%).

ASA = American Society of Anesthesiologists; BMI = body mass index; NYHA = New York Heart Association.

^a^ Smoking half a pack of cigarettes per day for at least 2 years.

^b^Two drinks or more daily, or weekly consumption of the equivalent of 150 ml of alcohol.

**Table 2:** Intraoperative variables.

	CTRL Group (N = 29)	DEX Group (N = 28)
Duration of anesthesia (hour)	6.0 ± 2.0	5.7 ± 2.3
Dosage of anesthetics		
Propofol (mg)	1326 ± 757	1235 ± 596
Remifentanil (μg)	2470 ± 1570	2186 ± 1035
Morphine before end of surgery (mg)	6.6 ± 1.2	6.5 ± 1.0
Intraoperative use of ondansetron	14 (48.3%)	11 (39.3%)
Intraoperative use of glucocorticoids	21 (72.4%)	16 (67.9%)
Duration of surgery (hour)	4.5±1.9	4.3±2.0
Type of surgery		
Radical gastrectomy	8 (27.6%)	8 (28.6%)
Radical resection for colorectal cancer	9 (31.0%)	13 (46.4%)
Whipple operation	7 (24.1%)	3 (10.7%)
Hepatectomy for liver cancer	2 (6.9%)	2 (7.1%)
Cholecystectomy and T-tube drainage	3 (10.3%)	2 (7.1%)
Total intraoperative infusion (ml)^a^	2805 (2368, 3242)	2738 (2342, 3134)
Estimated intraoperative bleeding (ml)	150 (72, 228)	137 (63, 212)
Duration of PCIA (hour)	68.3± 8.2	70.5±4.9

Results are presented as mean ± SD, number (%) or median (interquartile range).

^a^ Including lactate ringer’s fluid, succinylated gelatin and hydroxyethyl starch.

### Efficacy outcomes

**Fig 2:**
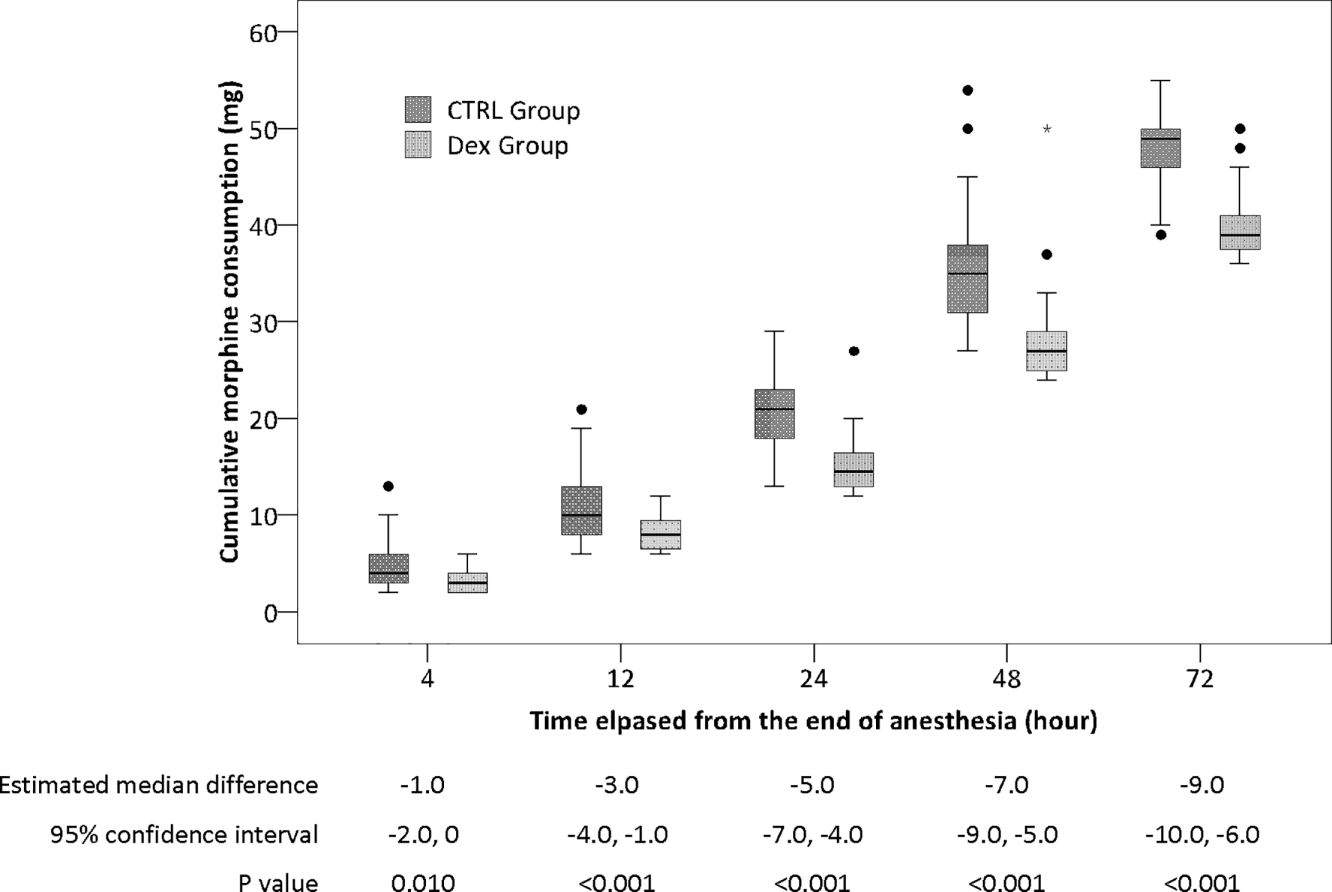
Cumulative consumption of morphine at different time-points after surgery. The differences between two groups were significant at 12, 24, 48 and 72 hours after surgery. The box and whiskers plots show medians, interquartile ranges and outer ranges (SPSS 14.0, SPSS, Inc., Chicago, IL). Bonferroni method was used to control type I error and P value less than 0.010 was considered to be statistical significant. DEX = dexmedetomidine group; CTRL = control group.

The mean (± SD) consumption of dexmedetomidine in the DEX group was 159.1 ± 2.8 μg. The 72-hour morphine consumption was lower in the DEX group than in the CTRL group (median 39.0 mg [interquartile range 37.3, 41.0] with DEX vs. 49.0 mg [45.5, 50.0] with CTRL; median difference -9.0 mg [95% CI -10.0, -6.0], P < 0.001). The differences of cumulative morphine consumption between the two groups were significant at 12, 24, 48, and 72 hours after surgery (Table 3, Fig 2).

**Table 3:** Efficacy outcomes.

	CTRL group (N = 29)	DEX group (N = 28)	Median difference or OR (95% CI)	P value
**Primary outcome**				
72-hour morphine consumption (mg)	49.0 (45.5, 50.0)	39.0 (37.3, 41.0)	-9.0 (-10.0, -6)	< 0.001
**Secondary outcomes**				
Number of patients with rescue morphine	9 (31.0%)	2 (7.1%)	0.171 (0.033, 0.881)	0.041
Dosage of rescue morphine (mg)	0.0 (0.0, 2.0)	0.0 (0.0, 0.0)	0.0 (0.0, 0.0)	0.049
NRS pain score after surgery, at rest (score)				
4 hours	4.0 (3.0, 4.5)	2.0 (2.0, 3.0)	-1.0 (-2.0, -1.0)	< 0.001
12 hours	4.0 (3.0, 5.0)	2.0 (2.0, 3.0)	-2.0 (-2.0, -1.0)	< 0.001
24 hours	4.0 (3.0, 5.0)	2.0 (1.0, 4.0)	-1.0 (-2.0, 0.0)	0.007
48 hours	4.0 (3.0, 5.0)	2.0 (1.0, 3.5)	-2.0 (-2.0, -1.0)	< 0.001
72 hours	2.0 (1.0, 3.0)	1.0 (0.3, 2.0)	-1.0 (-2.0, 0.0)	0.042
NRS pain score after surgery, with movement (score)				
4 hours	4.0 (4.0, 4.5)	3.0 (3.0, 4.8)	-1.0 (-2.0, 0.0)	0.002
12 hours	4.0 (3.0, 5.0)	4.0 (3.0, 5.0)	0.0 (-1.0, 0.0)	0.505
24 hours	5.0 (4.0, 6.0)	3.0 (2.3, 5.0)	-1.0 (-2.0, 0.0)	0.004
48 hours	5.0 (4.0, 6.0)	3.0 (2.0, 4.8)	-2.0 (-3.0, -1.0)	< 0.001
72 hours	3.0 (1.5, 4.5)	2.0 (1.0, 2.0)	-1.0 (-2.0, 0.0)	0.012
Supplemental use of flurbiprofen axetil	13.0 (44.8%)	6.0 (21.4%)	0.3 (0.1, 1.1)	0.061
NRS score of sleep quality (score)				
The night of surgery	5.0 (3.0, 6.0)	6.5 (4.3, 8.8)	2.0 (0.0, 3.0)	0.031
The 1^st^ night after surgery	7.0 (6.0, 7.0)	8.0 (7.0, 9.0)	2.0 (1.0, 2.0)	< 0.001
The 2^nd^ night after surgery	8.0 (7.0, 9.0)	8.0 (8.0, 9.0)	0.0 (0.0, 1.0)	0.471
**Other outcomes**				
Incidence of postoperative complications (n)	9 (31.0%)	3 (10.7%)	0.267 (0.064, 1.117)	0.060
Postoperative complications (n)				
Acute coronary syndrome	5 (17.2%)	1 (3.6%)	0.178 (0.019, 1.631)	0.194
New onset atrial fibrillation	1 (3.4%)	0 (0.0%)	0.966 (0.901, 1.034)	> 0.999
Transient ischemic attack	2 (6.9%)	0 (0.0%)	0.931 (0.843, 1.028)	0.491
Delirium	2 (6.9%)	0 (0.0%)	0.931 (0.843, 1.028)	0.491
Urinary tract infection	1 (3.4%)	0 (0.0%)	0.966 (0.901, 1.034)	> 0.999
Intraabdominal infection	1 (3.4%)	0 (0.0%)	0.966 (0.901, 1.034)	> 0.999
Incomplete ileus	0 (0.0%)	2 (7.1%)	1.077 (0.972, 1.193)	0.237
ICU admission after surgery	7 (24.1%)	11 (39.3%)	2.034 (0.651, 6.356)	0.219
Length of stay in hospital after surgery (day)	13.0 (10.4, 15.6)	12.0 (11.0, 13.0)	0.644 (0.366, 1.134)	0.427
30-day mortality	0 (0.0%)	0 (0.0%)	-	-

Results are presented as median (interquartile range) or number (%).

The NRS pain scores at rest were lower in the DEX group than in the CTRL group at 4, 12, 24, and 48 hours after surgery. The NRS pain scores with movement were lower in the DEX group than in the CTRL group at 4, 24, and 48 hours after surgery. The NRS score of subjective sleep quality was higher (better) in the DEX group than in the CTRL group during the night of surgery and the 1^st^ night after surgery (P = 0.031 and P < 0.001, respectively). The incidence of postoperative complications was not statistically different between groups, although it was slightly lower in the DEX group (P = 0.060). No patient died within 30 days (Table 3).

### Safety outcomes

The incidence of PONV was lower in the DEX group than in the CTRL group (10.7% vs. 37.9%, P = 0.029). The incidences of emergence agitation, tachycardia, hypotension, and hypertension did not differ between groups. No patient developed bradycardia, over sedation or respiratory depression during the study period (Table 4).

**Table 4:** Safety outcomes.

	CTRL group (N = 29)	DEX group (N = 28)	P value
Emergence agitation	0 (0.0%)	1 (3.6%)	0.491
Bradycardia	0 (0.0%)	0 (0.0%)	-
Tachycardia	1 (3.4%)	0 (0.0%)	> 0.999
Hypotension	2 (6.9%)	1 (3.6%)	> 0.999
Hypertension	8 (27.6%)	7 (25.0%)	> 0.999
Over sedation	0 (0.0%)	0 (0.0%)	-
Respiratory depression	0 (0.0%)	0 (0.0%)	-
Postoperative nausea and vomiting	11 (37.9%)	3 (10.7%)	0.029

## Discussion

The present study found that, in elderly patients after open abdominal surgery, combined use of dexmedetomidine and morphine for PCIA decreases morphine consumption. This combination also improves analgesic effects, ameliorates sleep quality and reduces the occurrence of PONV, without increasing adverse events.

When used for postoperative analgesia, the dose or infusion rate of dexmedetomidine varied widely. In a study of patients after hysterectomy, dexmedetomidine (at a rate of 0.02μg/kg/h) in combination with sufentanil reduced 72-hour sufentanil consumption by 30% [18]. In another study of patients after Caesarean section, combined use of dexmedetomidine (0.045μg/kg/h) and sufentanil for PCIA decreased 24-hour sufentanil consumption from 54.5 mg to 43.9 mg [15]. In present study, the background infusion rate of dexmedetomidine was set at 2 μg/h (i.e., about 0.02–0.04 μg/kg/h according to the body weight of enrolled patients). This infusion rate was similar to above studies [15,18] but lower than others (varied from 0.08 μg/kg/h to 0.5 μg/kg/h, for durations of 24 to 48 hours) [14–16]. We adopted a lower background infusion rate because of the following considerations. First, infusion rate of 0.02–0.045 μg/kg/h was verified to be effective in reducing opioid consumption [15,18]. Secondly, patients enrolled in the present study were older (mean age 66.8 years) than in previous ones (range 30–58 years old) [11,14–18]. Thirdly, the expected duration of dexmedetomidine infusion was 72 hours in the present study, longer than in previous ones (24 to 48 hours) [11,14–18]. Therefore, we chose a lower dose in order to decrease the potential dose-related side effects.

In line with previous studies [11,14–18], our results also showed that combined use of dexmedetomidine reduces morphine consumption and improves analgesic effect in patients after abdominal surgery. It should be noted that, in previous studies, the reduction of 24-hour opioid consumption varied from 22% to 41% with the use of dexmedetomidine [11,14–18]. In present study, use of dexmedetomidine reduced 24-hour morphine consumption by 22.3%, within the lower range of the previously results. The reason may be that the dose of dexmedetomidine was relatively low in our study than in previous ones. For example, in previous studies, dexmedetomidine at (mean or estimated) doses of 576 μg [11], 207.4 μg [16], and 116.5 μg [14] reduced 24-hour opioid consumptions by 41%, 40.7%, and 29%, respectively. In the present study, a mean dose of 60.1 μg dexmedetomidine was administered during the first 24 hours. In a recent study, Gao et al [28] reported that 100 μg dexmedetomidine reduced 24-hour sufentanil consumption by 12.3%; the opioid-sparing effect was less than our study. Decreased morphine consumption might have benefit in lowering the incidence of opioid side effect, such as PONV [11, 14, 16, 28]. In present study, we also noticed a reduction in incidence of PONV in the DEX group.

It was reported that continuous infusion of low-dose dexmedetomidine (at a rate of 0.1 μg/kg/h) during the night of surgery improves sleep structure (by decreasing stage N1 sleep, increasing stage N2 sleep and sleep efficiency) and subjective sleep quality [20–21]. In present study, dexmedetomidine infusion improved postoperative sleep quality during the night of surgery and the 1^st^ night after surgery. This result was similar to those in our previous studies [20–21]. However, we did not monitor polysomnography and observe its effect on sleep efficiency and architecture. In a recent study, Chen et al. [29] also found that combined use of dexmedetomidine with sufentanil for PCIA improves postoperative sleep quality.

Dexmedetomidine administered by PCIA might be helpful to improve postoperative outcome [20–21]. In present study, the incidence of complications was slightly lower (although not statistically significant) in the DEX group after surgery. Considering the small sample size of the present study, a well-designed large sample size trial is needed to further elucidate this problem in geriatric patients. Major concerns in using dexmedetomidine for postoperative analgesia were potential side effects such as bradycardia, hypotension and over sedation [11, 30]. However, our results did not find any difference in the occurrence of adverse events between groups.

The present study had several limitations. First, as a pilot study, the sample size was small and might have underestimated the incidence of adverse events produced by dexmedetomidine. Second, we only enrolled patients after open abdominal surgery; this might restrict the generalizability of our results. However, patients after open abdominal surgery represent one of the most painful population. Third, we did not monitor polysomnogram after surgery; and, therefore, did not know whether dexmedetomidine administered by PCIA could also improve sleep architecture. However, our results provide clues for further investigation.

## Conclusions

For elderly (≥ 60 years) patients after open abdominal surgery, dexmedetomidine in combination with morphine for PCIA decreases morphine consumption, improves analgesic effects and subjective sleep quality without increasing adverse events.

## Supporting information

S1 File PLOSOne_Clinical_Studies_Checklist. (DOCX)

S2 File Study-protocol-Chinese. (DOCX)

S3 File Study-protocol-English. (DOCX)

S4 File IRB approval file. (PDF)

S5 File IRB approval file English translation. (DOCX)

S6 File Trial data. (XLSX)

S7 File CONSORT 2010 Checklist. (DOC)
