# Supplementary material for: Dexmedetomidine in combination with morphine improves postoperative analgesia and sleep quality in elderly patients after open abdominal surgery: A pilot randomized control trial
Source: PLoS One. 2018 Aug 14;13(8):e0202008. doi: 10.1371/journal.pone.0202008 (PMC6091958; doi:10.1371/journal.pone.0202008)
Supplement: S2 File — (DOCX) [file pone.0202008.s002.docx]

**右美托咪定用于老年患者术后镇痛的有效性研究**

**研究方案**

**版本号：201406-1.1**

**日 期：2014-10-09**

**申请人：王东信**

**科室：麻醉科**

**单位：北京大学第一医院**

**研究背景**

减轻外科术后患者的疼痛是麻醉科医生的职责，也是患者享有的基本权利。目前病人自控静脉镇痛仍是术后急性疼痛治疗的主要方式之一 [1]。

阿片类药物仍然是围术期镇痛的一线药物，但是仅使用阿片类药物进行镇痛并不能达到良好的镇痛效果，而且随着药物剂量的增加副作用也会明显增加，如呼吸抑制、恶心呕吐等，多模式镇痛是解决这一临床问题的方法之一。多模式镇痛的关键在于通过多种镇痛药物或镇痛方式的组合，发挥各种镇痛方式的优势并降低各种镇痛方式的副作用，右美托咪定是临床可选择的辅助药物之一。

一、右美托咪定的药理学特性

右美托咪定为美托咪定的活性右旋异构体，具有抗交感、镇静和镇痛的作用，与美托咪定相比，本品对中枢α2-肾上腺素受体激动的选择性更强，对α2-肾上腺素受体是可乐定的8倍。

在介导本品的主要药理和治疗效应中，α2A受体亚型起着重要作用，α2A受体存在于突触前和突触后，主要涉及抑制[去甲肾上腺素](http://baike.baidu.com/view/218098.htm)的释放和神经元的兴奋。本品通过激动突触前膜α2受体，抑制了去甲肾上腺素的释放，并终止了疼痛信号的传导；通过激动突触后膜受体，右美托咪定抑制了交感神经活性从而引起血压和心率的下降；与脊髓内的α2受体结合产生镇痛作用时，可导致镇静及焦虑缓解。本品还能降低麻醉剂的用药剂量，改善手术中血液动力学的稳定性和降低心肌局部缺血的发生率。

静脉输注后，右美托咪定的药代动力学参数如下：快速分布相的分布半衰期（t1/2）大约为6分钟；终末清除半衰期（t1/2）大约为2小时；稳态分布容积（Vss）大约为118升。清除率大约为39L/h。评价清除率的平均体重为72kg。

右美托咪定几乎完全被生物转化，极少以原形从尿和粪便中排出。生物转化包括直接[葡萄](http://baike.baidu.com/view/5119.htm)苷酸化和[细胞色素P450](http://baike.baidu.com/view/2564945.htm)介导的代谢。右美托咪定的主要代谢途径是：直接N-葡萄苷酸化成非活性代谢产物；脂肪羟基化作用（主要由CYP2A6介导）产生3-羟基右美托咪定、3-羟基右美托咪定葡糖苷酸和3-羧基右美托咪定；右美托咪定N-甲基化产生3-羟基N-甲基右美托咪定、3-羧基N-甲基右美托咪定和N-甲基O-葡糖苷酸右美托咪定。

右美托咪定的终末清除半衰期（t1/2）大约为2小时，清除率大约为39L/h。质量平衡研究证实静脉输注放射性标记的右美托咪定9天后平均95%的放射活性物质从尿中回收，4%在粪便中。尿中可以检测到右美托咪定原形。输注本品后24小时内大约85%的放射活性物质从尿中排出。尿中排出的放射活性物质分段分离证实为N-葡萄苷酸化产物占34%。另外，脂肪羟基化作用产物3-羟基右美托咪定、3-羟基右美托咪定葡糖苷酸和3-羧酸右美托咪定大约占14%。右美托咪定N-甲基化产生的3-羟基N-甲基右美托咪定、3-羧基N-甲基右美托咪定和N-甲基O-葡糖苷酸右美托咪定大约占18%。N-甲基代谢产物本身是次要循环成分，在尿中未检测到。大约28%的尿代谢物未被识别。

二、右美托咪定用于术后镇痛的临床研究证据

Lin TF等人[1]的研究纳入了100例接受子宫切除手术的患者。研究人员将患者随机分为两组：吗啡组（1mg/ml）和右美托咪定组（吗啡1mg/ml+右美托咪定5μg/ml）。镇痛泵设置为每次追加剂量为1ml，间隔时间为5分钟，没有背景剂量。结果显示，右美托咪定可以减少吗啡用量达30%，且患者的疼痛评分和恶心呕吐的发生率显著低于吗啡组患者。

YuyanNie等人的研究[2]也发现剖宫产术后患者在术后使用右美托咪定+舒芬太尼复合方案（右美托咪定3μg/ml+舒芬太尼1μg/ml，持续输注1ml/h，单次追加剂量2ml，锁定时间间隔8min）较单纯使用舒芬太尼方案（1μg/ml，持续输注1ml/h，单次追加剂量2ml，锁定时间间隔8min）可以显著减少术后舒芬太尼的用量和降低术后恶心呕吐的发生率。

有研究显示，术后24小时内给予输注右美托咪定可以有效改善胸科手术后患者的镇痛效果,并使48小时内阿片类药物的使用量降低40%[3]。

三、已有临床研究的不足之处

但是以上研究存在一定的不足。第一，上述3项研究的患者年龄平均约为30-40岁，缺乏老年患者的有效性和安全性资料。已有的研究显示，老年患者更容易出现右美托咪定相关的药物不良反应，如窦性心动过缓、低血压和高血压等 [4]。第二，上述研究仅使用了24小时，而一般临床实践中术后镇痛使用3天，因此以上研究观察时间过短，而长时间使用右美托咪定有可能会增加药物不良反应的发生率 [5]。第三，上述研究均缺少关于患者围术期安全性指标的观察和报道，因此我们认为需要进一步通过研究严密观测安全性数据 [1-3]。

我们推测右美托咪定可以改善老年患者术后镇痛的效果并减少阿片类药物的使用剂量，但是其有效性和安全性还需要进一步的研究。

参考文献

1. [Lin TF](http://www.ncbi.nlm.nih.gov/pubmed?term=Lin%20TF%5BAuthor%5D&cauthor=true&cauthor_uid=18987053), [Yeh YC](http://www.ncbi.nlm.nih.gov/pubmed?term=Yeh%20YC%5BAuthor%5D&cauthor=true&cauthor_uid=18987053), [Lin FS](http://www.ncbi.nlm.nih.gov/pubmed?term=Lin%20FS%5BAuthor%5D&cauthor=true&cauthor_uid=18987053), [Wang YP](http://www.ncbi.nlm.nih.gov/pubmed?term=Wang%20YP%5BAuthor%5D&cauthor=true&cauthor_uid=18987053), [Lin CJ](http://www.ncbi.nlm.nih.gov/pubmed?term=Lin%20CJ%5BAuthor%5D&cauthor=true&cauthor_uid=18987053), [Sun WZ](http://www.ncbi.nlm.nih.gov/pubmed?term=Sun%20WZ%5BAuthor%5D&cauthor=true&cauthor_uid=18987053), [Fan SZ](http://www.ncbi.nlm.nih.gov/pubmed?term=Fan%20SZ%5BAuthor%5D&cauthor=true&cauthor_uid=18987053). Effect of combining dexmedetomidine and morphine for intravenous patient-controlled analgesia. Br J Anaesth. 2009 Jan; 102(1): 117-22.
2. [Nie Y](http://www.ncbi.nlm.nih.gov/pubmed?term=Nie%20Y%5BAuthor%5D&cauthor=true&cauthor_uid=24463478)1, [Liu Y](http://www.ncbi.nlm.nih.gov/pubmed?term=Liu%20Y%5BAuthor%5D&cauthor=true&cauthor_uid=24463478), [Luo Q](http://www.ncbi.nlm.nih.gov/pubmed?term=Luo%20Q%5BAuthor%5D&cauthor=true&cauthor_uid=24463478), [Huang S](http://www.ncbi.nlm.nih.gov/pubmed?term=Huang%20S%5BAuthor%5D&cauthor=true&cauthor_uid=24463478). Effect of dexmedetomidine combined with sufentanil for post-caesarean section intravenous analgesia: a randomised, placebo-controlled study. Eur J Anaesthesiol. 2014 Apr; 31(4): 197-203.
3. Michael A. E, et al. Dexmedetomidine infusion for analgesia up to 48 hours after lung surgery performed by lateral thoracotomy. Proc (BaylUniv Med Cent) 2014; 27(1):3–10.
4. [Park SH](http://www.ncbi.nlm.nih.gov/pubmed?term=Park%20SH%5BAuthor%5D&cauthor=true&cauthor_uid=24910729), [Shin YD](http://www.ncbi.nlm.nih.gov/pubmed?term=Shin%20YD%5BAuthor%5D&cauthor=true&cauthor_uid=24910729), [Yu HJ](http://www.ncbi.nlm.nih.gov/pubmed?term=Yu%20HJ%5BAuthor%5D&cauthor=true&cauthor_uid=24910729), [Bae JH](http://www.ncbi.nlm.nih.gov/pubmed?term=Bae%20JH%5BAuthor%5D&cauthor=true&cauthor_uid=24910729), [Yim KH](http://www.ncbi.nlm.nih.gov/pubmed?term=Yim%20KH%5BAuthor%5D&cauthor=true&cauthor_uid=24910729).Comparison of two dosing schedules of intravenous dexmedetomidine in elderly patients during spinal anesthesia.Korean J Anesthesiol. 2014 May;66(5):371-6.
5. [Ozaki M](http://www.ncbi.nlm.nih.gov/pubmed?term=Ozaki%20M%5BAuthor%5D&cauthor=true&cauthor_uid=23912755)1, [Takeda J](http://www.ncbi.nlm.nih.gov/pubmed?term=Takeda%20J%5BAuthor%5D&cauthor=true&cauthor_uid=23912755), [Tanaka K](http://www.ncbi.nlm.nih.gov/pubmed?term=Tanaka%20K%5BAuthor%5D&cauthor=true&cauthor_uid=23912755), et al. Safety and efficacy of dexmedetomidine for long-term sedation in critically ill patients.JAnesth. 2014 Feb;28(1):38-50.

**研究目的**

本研究的主要目的是观察右美托咪定辅助吗啡静脉镇痛的效果，为临床术后镇痛管理提供进一步的资料，改善临床术后镇痛的效果。

1. 主要观察终点：术后72小时内阿片类药物的累积使用量
2. 次要观察终点：术后疼痛评分；术后住院期间的药物不良事件发生率；术后住院期间并发症发生率；术后30天存活率。

**研究设计**

本研究为前瞻性、随机、双盲、对照研究

**研究方案**

1. 本研究为前瞻性、随机、双盲对照研究。
2. 研究主要目标：两组患者术后疼痛评分；两组患者术后舒芬太尼的用量。
3. 研究次要目标：右美托咪定相关的药物不良反应。
4. 病人入选标准：
   1. 年龄大于60岁；
   2. 同意参加本研究的患者；
   3. 全身麻醉下接受腹部择期手术、术后行静脉镇痛的患者；
5. 病人排除标准：
   1. 术前精神分裂症
   2. 病态窦房结综合征
   3. 窦性心动缓 （<50次/分）
   4. Ⅱ度或Ⅲ度房室传导阻滞；
   5. 严重肝功能异常（Child-PughC级以上）
   6. 严重肾功能异常（术前接受透析）
   7. 体重小于50Kg
6. 中止及退出标准
7. 受试者自愿退出
8. 出现严重不良反应且研究主管机构认为需要研究中止时。
9. 随机原则和盲法
10. 采用电脑产生的区组随机数据。
11. 随机数据由专人保管（研究协调员），所产生的随机数据装入信封内，按照患者的入选顺序分配随机数据。根据随机数据按1:1比例将患者分为两组。
12. 研究药物由指定的研究人员根据随机数据号码负责配置。使用品牌及外观统一的50ml注射器进行药物稀释，右美托咪定和生理盐水均为无色无味透明液体。配制药物的研究人员不参加麻醉、镇痛管理和病人随访。配制好的药物交给患者的主管麻醉医生。
13. 术中药物给予和术后镇痛泵由麻醉医师负责，术后疼痛评估和术后镇痛泵的使用情况由研究人员完成。
14. 在研究期间患者、负责麻醉/围术期管理的医务人员和负责随访的研究人员均不知道分组结果。
15. 干预措施
16. 根据随机分组结果，患者分别接受右美托咪定或生理盐水。
17. 试验药物的干预方案。
    1. 在患者转出PACU时开始给予病人自控静脉镇痛。
    2. 自控镇痛泵的设置为持续剂量1ml/h，单次追加剂量为2ml，追加间隔时间为8min。总剂量均为100ml。
    3. 对照组配方为吗啡0.5mg/ml；试验组配方为吗啡0.5mg/ml+右美托咪定2μg/ml。

9.研究实施流程

- 1. 术前1～2天访视病人，获得知情同意书后，收集基本资料。
  2. 所有患者均不接受术前镇静药物，入室后连接心电图、无创血压及脉搏样饱和度监测，必要时可监测直接动脉血压或者中心静脉压。

1. 两组患者均接受全凭静脉全身麻醉，术后进入麻醉恢复室，符合转出标准后由麻醉医师及外科医师共同转送至普通病房。
2. 患者入室后记录生命体征等基本资料；
3. 麻醉诱导：舒芬太尼0.1-0.3μg/Kg，异丙酚2-4μg/ml（效应室靶控），爱可松0.6-0.8mg/Kg；
4. 麻醉维持：异丙酚 1-3μg/ml（效应室靶控），单次追加顺式阿曲库铵0.02-0.04mg/Kg维持肌肉松弛，瑞芬太尼2-5ng/ml（效应室靶控），麻醉深度维持在BIS值40-60之间；手术结束前30分钟给予吗啡0.1mg/Kg抑制瑞芬诱导的疼痛过敏反应。
5. 患者在手术结束并苏醒后进入术后恢复室（PACU），每隔15分钟评估一次患者疼痛评分，如果患者NRS评分>3，则可以单次给予吗啡2-4mg，直至NRS低于3。
6. 患者满足出PACU标准后可转入普通病房，并开始接受病人自控静脉镇痛。
7. 患者在转入普通病房后接受连续72小时的术后生命体征监测，血压，心率，氧和和心电图。
8. 术后连续3天评估患者的疼痛评分。
9. 记录患者在住院期间的药物不良反应、并发症和死亡率。
   - 1. 疼痛评估

疼痛评估采用数字疼痛评分（NRS），评估患者在静息和活动时的疼痛评分。患者麻醉苏醒后每15分钟评估一次疼痛评分，可间断给予吗啡1-2mg，使患者NRS评分低于3分。记录患者术后4，12，24，48和72小时静息和活动时疼痛评分。

- - 1. 镇痛补救方案

若患者在转运至普通病房后出现NRS大于4分，首选方案是追加病人自控镇痛的单次追加剂量，也可以间断给予吗啡2-4mg，直至NRS低于3分。

12. 术后不良事件观察

1. 术后连续监测患者的心电图、心率、血压和脉搏氧饱和度72小时（由临床医师下长期医嘱，病房护士执行）。
2. 为避免遗漏重要数据，随访人员每隔12小时利用心电监护仪的回放功能对数据进行复习，详细记录有异常的生命体征发生时间、持续时间和临床治疗方案。
3. 向病房医生及护士详细说明患者可能出现的不良反应和处理预案。当患者出现不良反应时，由病房医生直接处理或者通知研究随访人员进行处理，任何治疗措施都应进行记录。
4. 患者出现窦缓(心率<50次/分)时，可给予阿托品0.2-0.4mg静脉注射（必要时5-10分钟后可重复）。如果连续3次治疗后心率未超过50次/分，病房医生可终止试验药物输注并通报研究人员。
5. 患者出现心动过速HR>120bpm或HR增加超过基础值30%时，如果患者存在容量不足可给与300-500ml晶体液扩容；在容量充足情况下，可给予艾司洛尔20-50mg，间隔10分钟追加一次，若3次治疗后仍为心动过速，停止使用试验药物。
6. 患者出现收缩压小于90mmHg或血压降低大于基础血压的20％时，评估患者是否存在容量不足，可给与250ml晶体液进行扩容，必要时重复；给予麻黄素6mg静脉注射（必要时5-10分钟后可重复）或给予多巴胺静脉输注。如果经上述处理血压仍低，病房医生可停止输注试验药物并通报研究人员。
7. 患者出现高血压收缩压>180 mmHg或舒张压>100 mmHg或较基础值升高超过20%时，可给与盐酸乌拉地尔10-15mg或尼卡地平0.2-0.5mg静脉注射（必要时5-10分钟后重复），或持续输注上述药物。如果经上述处理后效果仍不明显，病房医生可停止输注试验药物并通报研究人员。
8. 患者出现缺氧SpO2 <90% 或较基线降低10%，可给予鼻导管吸氧（5L/min）、鼓励病人深呼吸/咳嗽。如果经上述处理后仍存在低氧血症，加大吸入氧流量或改为储氧面罩，病房医生可并停止使用试验药物并通报研究人员。
9. 患者出现呼吸抑制呼吸频率<8次/分或较基础值降低25%或者过度镇静（RASS评分<3分），病房医生可停止使用试验药物并通报研究人员。
10. 患者出现其他怀疑与试验药物可能有关的不良事件时，病房医生可终止试验药物并通知研究人员。
11. 任何情况下如试验药物被终止，研究人员应提供不含研究药物的镇痛泵继续完成术后镇痛。
12. 患者若出现恶心呕吐现象，可给予昂丹司琼5mg进行治疗。

13. 当患者出现严重不良事件时，由研究人员在24小时内通知研究负责人并上报临床伦理委员会。

**统计分析**

1. 样本量估计：我们之前的研究显示外科术后患者三天的吗啡总用量约为50mg [1]，根据Lin等人的研究术后使用右美托咪定+吗啡复合方案可以使患者术后24小时内吗啡的使用剂量降低约30%。由于我们在本次研究中以老年患者为观察对象且减少了右美托咪定的剂量，因此我们假设在本研究中试验组可以较对照组的72小时吗啡总用量降低约20% （50±12mg vs.40±12mg），检验效能为0.8，检验水准为0.05，每组约需要24例患者，考虑到20%的失访率，预计每组需要29例患者，两组共计58例。
2. 统计学分析。
   1. 主要观察指标的分析

术后72小时内吗啡用量采用独立样本t检验。

- 1. 次要观察指标的分析

疼痛评分采用重复测量资料的F检验。

药物不良反应、并发症发生率和死亡率采用卡方检验。

- 1. 缺失资料的处理。

缺失的资料按照研究方案相关定义中的最差值进行赋值。
