# Supplementary material for: Dexmedetomidine in combination with morphine improves postoperative analgesia and sleep quality in elderly patients after open abdominal surgery: A pilot randomized control trial
Source: PLoS One. 2018 Aug 14;13(8):e0202008. doi: 10.1371/journal.pone.0202008 (PMC6091958; doi:10.1371/journal.pone.0202008)
Supplement: S3 File — (DOCX) [file pone.0202008.s003.docx]

**Dexmedetomidine in combination with morphine improves postoperative analgesia and sleep quality in elderly patients after open abdominal surgery:**

**a pilot randomized control trial**

**Study Protocol**

Version: **201406-1.1**

Date: **2014-10-09**

Primary investigator: Wang Dongxin

Department: Department of Anesthesiology and Critical Care

Hospital: Peking University First Hospital

**Background**

Postoperative analgesia is the responsibility of anesthesiologist and it’s also a reserved right of patient. Patient-controlled analgesia(PCA) is the main method for pain management [1].

Opioids are the mainstay of perioperative analgesia; however, 45% to 86% of patients reported moderate to severe pain after surgery and up to 80% of patients suffer from adverse effects of opioids [1-3]. Multimodal analgesia is recommended to reduce opioid consumption and related adverse events [4]. As an alternative, dexmedetomidine may be used as an analgesic and antinociceptive adjuvant in pain management in postoperative period.

1. **The pharmacodynamics of dexemedetomidine**

Dexmedetomidine is a potent α2 adrenoceptor agonist with an 8 times greater affinity to α2 adrenoceptors than clonidine. The α2 adrenoceptor agonists are known to have sedative, anxiolytic, analgesic, and anesthetic-sparing effect.

In general, presynaptic activation of the α2adrenoceptor inhibits the release of norepinephrine, terminating the propagation of pain signals. Postsynaptic activation of α2adrenoceptors in the central nervous system (CNS) inhibits sympathetic activity and thus can decrease blood pressure and heart rate. Combination of these effects, dexmedetomidine can produce analgesia, sedation, and anxiolysis. As an α2 agonists, dexmedetomidine have several advantages during the perioperative period. It reduces anesthetic and opioid requirements; attenuates of the neuroendocrine and hemodynamic responses to anesthesia and surgery and offers benefits in the prophylaxis and adjuvant treatment of perioperative myocardial ischemia.

Intravenous dexmedetomidine exhibits linear pharmacokinetics with a rapid distribution half-life of approximately 6 minutes and a terminal elimination half-life of approximately 2 hours.

Plasma protein binding of dexmedetomidine is about 94% (mostly albumin). The steady-state volume of distribution is 118 L. Clearance is estimated to be approximately 39 L/h. The mean body weight associated with this clearance estimate was 72 kg.

Dexmedetomidine undergoes almost complete biotransformation through direct glucuronidation and cytochrome P450 metabolism, all hepatic processes, with very little excretion of unchanged molecules in the urine or feces. Direct N-glucuronidation by uridine 5′-diphospho-glucuronosyltransferase accounts for about 34% of dexmedetomidine metabolism. In addition, hydroxylation mediated by cytochrome P450 (CYP) enzymes (mainly CYP2A6) was demonstrated in human liver microsomes.

A terminal elimination half-life of approximately 2 hours and clearance is estimated to be approximately 39 L/h. Its metabolites include 3-hydroxy, 3-carboxy, 3-hydroxy N-methyl, 3-carboxy N-methyl, and N-methyl O-glucuronide dexmedetomidine.

1. **The clinical evidence of the applications of dexmedetomidine**

In a recent study, 100 women undergoing abdominal total hysterectomy were allocated to receive either morphine 1 mg/ml alone (Group M) or morphine 1 mg/ml plus dexmedetomidine 5 mg/ml (Group D) for postoperative intravenous PCA, which was programmed to deliver 1 ml per demand with a 5 min lockout interval without background infusion. The result showed that the addition of dexmedetomidine to morphine resulted in significant reduction in morphine consumption (about 29%) and less incidence of PONV.

Nie and colleagues recruited one hundred and twenty patients scheduled for elective caesarean delivery under spinal anesthesia.  They were randomly allocated into three groups. Group 1: normal saline bolus after delivery and sufentanil PCA; Group 2: dexmedetomidine bolus (0.5 μg /kg) after delivery and sufentanil PCA; Group 3: dexmedetomidine bolus (0.5 μg /kg) after delivery and sufentanil with dexmedetomidine PCA (background infusion of 0.045 μg/kg /h with a bolus of 0.07 μg /kg). They reported that the combination of sufentanil and dexmedetomidine for PCA after caesarean section can reduce sufentanil consumption and improve patients' satisfaction.

Another study showed that the addition of dexmedetomidine to the pain management protocol for post-thoracotomy patients may be safe and equal analgesia with a 41% deceasing in opioid use in the dexmedetomidine group in 48 hours.

1. **The limitation of the previous studies**

Firstly, most of the studies were performed in young patients. Data in elderly patients are insufficient. Side effects due to dexmedetomidine such as hypotension, bradycardias and hypertension are more common in eldly patients. Second, dexmedetomidine administration was usually ended at about 24 hours after surgery in the present study.While, patient-controlled intravenous analgesia (PCIA) was provided for all patientsfrom end of anesthesia until 72 hours after surgery in clinical practice. The prolonged use of dexmedetomidine may result in side effect. Thirdly, safety outcomes werenot reported in detail in the majority of available studies.

We hypothesized that combined dexmeditomidine and morphine analgesia may decreases morphine consumption and improves analgesic effects.The efficacy and safety need more research.

**Reference**

1. [Lin TF](http://www.ncbi.nlm.nih.gov/pubmed?term=Lin%20TF%5BAuthor%5D&cauthor=true&cauthor_uid=18987053), [Yeh YC](http://www.ncbi.nlm.nih.gov/pubmed?term=Yeh%20YC%5BAuthor%5D&cauthor=true&cauthor_uid=18987053), [Lin FS](http://www.ncbi.nlm.nih.gov/pubmed?term=Lin%20FS%5BAuthor%5D&cauthor=true&cauthor_uid=18987053), [Wang YP](http://www.ncbi.nlm.nih.gov/pubmed?term=Wang%20YP%5BAuthor%5D&cauthor=true&cauthor_uid=18987053), [Lin CJ](http://www.ncbi.nlm.nih.gov/pubmed?term=Lin%20CJ%5BAuthor%5D&cauthor=true&cauthor_uid=18987053), [Sun WZ](http://www.ncbi.nlm.nih.gov/pubmed?term=Sun%20WZ%5BAuthor%5D&cauthor=true&cauthor_uid=18987053), [Fan SZ](http://www.ncbi.nlm.nih.gov/pubmed?term=Fan%20SZ%5BAuthor%5D&cauthor=true&cauthor_uid=18987053). Effect of combining dexmedetomidine and morphine for intravenous patient-controlled analgesia. Br J Anaesth. 2009 Jan; 102(1): 117-22.
2. [Nie Y](http://www.ncbi.nlm.nih.gov/pubmed?term=Nie%20Y%5BAuthor%5D&cauthor=true&cauthor_uid=24463478)1, [Liu Y](http://www.ncbi.nlm.nih.gov/pubmed?term=Liu%20Y%5BAuthor%5D&cauthor=true&cauthor_uid=24463478), [Luo Q](http://www.ncbi.nlm.nih.gov/pubmed?term=Luo%20Q%5BAuthor%5D&cauthor=true&cauthor_uid=24463478), [Huang S](http://www.ncbi.nlm.nih.gov/pubmed?term=Huang%20S%5BAuthor%5D&cauthor=true&cauthor_uid=24463478). Effect of dexmedetomidine combined with sufentanil for post-caesarean section intravenous analgesia: a randomised, placebo-controlled study. Eur J Anaesthesiol. 2014 Apr; 31(4): 197-203.
3. Michael A. E, et al. Dexmedetomidine infusion for analgesia up to 48 hours after lung surgery performed by lateral thoracotomy. Proc (BaylUniv Med Cent) 2014; 27(1):3–10.
4. [Park SH](http://www.ncbi.nlm.nih.gov/pubmed?term=Park%20SH%5BAuthor%5D&cauthor=true&cauthor_uid=24910729), [Shin YD](http://www.ncbi.nlm.nih.gov/pubmed?term=Shin%20YD%5BAuthor%5D&cauthor=true&cauthor_uid=24910729), [Yu HJ](http://www.ncbi.nlm.nih.gov/pubmed?term=Yu%20HJ%5BAuthor%5D&cauthor=true&cauthor_uid=24910729), [Bae JH](http://www.ncbi.nlm.nih.gov/pubmed?term=Bae%20JH%5BAuthor%5D&cauthor=true&cauthor_uid=24910729), [Yim KH](http://www.ncbi.nlm.nih.gov/pubmed?term=Yim%20KH%5BAuthor%5D&cauthor=true&cauthor_uid=24910729).Comparison of two dosing schedules of intravenous dexmedetomidine in elderly patients during spinal anesthesia.Korean J Anesthesiol. 2014 May;66(5):371-6.
5. [Ozaki M](http://www.ncbi.nlm.nih.gov/pubmed?term=Ozaki%20M%5BAuthor%5D&cauthor=true&cauthor_uid=23912755)1, [Takeda J](http://www.ncbi.nlm.nih.gov/pubmed?term=Takeda%20J%5BAuthor%5D&cauthor=true&cauthor_uid=23912755), [Tanaka K](http://www.ncbi.nlm.nih.gov/pubmed?term=Tanaka%20K%5BAuthor%5D&cauthor=true&cauthor_uid=23912755), et al. Safety and efficacy of dexmedetomidine for long-term sedation in critically ill patients.JAnesth. 2014 Feb;28(1):38-50.

**Purpose of this study**

The purpose of this pilot study was to investigate the effects of combined dexmeditomidine and morphine on opioid consumption in elderly patients after open abdominal surgery.

1. Primary outcome: the 72-hour morphine consumption
2. Secondary outcomes: NRS pain score, the occurrence of adverse events after surgery , the occurrence of complications within 30 postoperative days and 30-day mortality.

**Study Design**

This study is a prospective, randomized, double-blinded and placebo-controlled pilot trial.

**Materials and Methods**

1. This study is a prospective, randomized, double-blinded and placebo-controlled pilot trial.

2. Primary outcome: Cumulative morphine consumption of two group at postoperative 72-hour.

3. Secondary outcome: postoperative pain intensity and drug-related side effects of dexmedetomidine

4. Inclusion criteria:

1. elderly patients (60 years or older)
2. agree to participated in the study
3. scheduled to undergo selective open abdominal surgery and agree to receive patient-controlled postoperative analgesia.

5. Exclusion criteria:

Patients who meet any of the following criteria will be excluded:

1. history of schizophrenia
2. presence of sick sinus syndrome
3. severe bradycardia (heart rate less than 50 beats per minute)
4. atrioventricular block before surgery(II°or III°)
5. preoperative severe hepatic disease (Child-Pugh grade 3 or above)
6. preoperative chronic renal failure (need dialysis)
7. bodyweight less than 50 kg or more than 90 kg

6. [Withdrawal Criteria](https://www.google.com.hk/url?sa=t&rct=j&q=&esrc=s&source=web&cd=1&ved=0CBoQFjAA&url=%68%74%74%70%3a%2f%2f%77%77%77%2e%6d%65%72%72%69%6d%61%63%6b%70%68%61%72%6d%61%2e%63%6f%6d%2f%73%6f%6c%75%74%69%6f%6e%73%2f%70%69%70%65%6c%69%6e%65%2f%6d%6d%2d%31%31%31%2f%70%61%74%69%65%6e%74%2d%77%69%74%68%64%72%61%77%61%6c%2d%63%72%69%74%65%72%69%61&ei=O5i7U5ycFsSHkQXCoYDYAw&usg=AFQjCNH0WyZL7pHRKdxZ256NCAW3fasglQ&bvm=bv.70138588,d.dGI&cad=rjt)

1. Patients may withdraw from the study at any time for any reason.
2. If a patient suffers an adverse event, which in the judgment of the investigator presents an unacceptable consequence or risk to the patient, the patient can be withdrawn from the study.

7. Randomization and blinding

1. Random numbers will be generated using software (SAS 9.2, SAS Institute Inc, Cary, NC).
2. During the study period, consecutively recruited patients will be randomly divided into two groups by a study coordinator who prepare the study drugs according to randomization number in the envelopes before surgery. The random numbers will be generated in a 1:1 ratio.
3. All study drugs will be contained in the same 50 ml colorless reservoir bags and administered by the same analgesia devices (GEMSTAR®; Hospira, Inc., Lake Forest, IL, USA). The study coordinator will not participate in anesthesia, perioperative care and postoperative follow-up of the patients.
4. Healthcare team members will be in charge of anesthesia management and postoperative analgesia. NRS pain score and morphine consumption will be recorded by investigators.
5. All patients, healthcare team members, and investigators for patient follow up will be blinded to study group allocation.

8. Interventions

1. Patients will be randomly assigned to receive either dexmeditomidine plus normal saline or merely normal saline for PCA.
2. Patient-controlled intravenous analgesia (PCIA) will be provided for all patients from end of anesthesia until 72 hours after surgery.
3. For patients in the control group, PCIA is established with 100ml of 0.5mg/ml morphine; while for those in the dexmedetomidine (DEX) group, it is established with 100 ml of 0.5mg/ml morphine plus 2µg/ml dexmedetomidine.

9.  Study procedure

1. Potential participants will be screened the day before surgery. For patients who meet the inclusion/exclusion criteria and have given the written informed consents, baseline data collection and preoperative assessment will be performed the day before surgery.
2. All the patients in the study will not receive any premedication. Electrocardiogram, non-invasive blood pressure and pulse oximeter will routinely be used for monitoring during anesthesia. Invasive arterial blood pressure and central venous pressure will be monitored if necessary.
3. Total intravenous anesthesia will be provided for all patients. Patients will be monitored in the post-anesthesia care unit for at least 30 minutes before being sent back to the general wards. Vital signs will be recorded before anesthesia.
4. Induction was achieved with propofol(2-4μg/ml), sufentanil(0.1-0.3μg/Kg) and rocuronium(0.6-0.8mg/Kg).
5. General nesthesia will be maintained with intravenous propofol(1-3μg/ml) and remifentanil(2-5ng/ml). Additional muscle relaxant (cisatracurium,0.02-0.04mg/Kg) will be administered when deemed necessary. Bispectral Index was maintained between 40 and 60 during surgery. Morphine 0.1mg/Kg will be administered 0.5 hour before the end of surgery in order to relieve remifentanil-induced hyperalgesia.
6. Patients will be monitored in the post-anesthesia care unit before being sent back to the general wards. NRS score will be evaluated every 15min in the post-anesthesia care unit. The target of postoperative analgesia will be to maintain a Numeric Rating Scale pain score at rest ≤ 3. For patients with a NRS pain score > 4, PCIA bolus will be firstly administered; in case of no significant improvement after three consecutive boluses, extra morphine 2-4 mg per time (maximum 0.1 mg/kg per hour) will be administered and repeated.
7. Patients will be sent to the ward when they meet the discharge criteria for PACU and continue to receive patient-controlled intravenous analgesia.
8. NRS pain score (at rest and with movement) will be assessed during the first 3 postoperative days.
9. Adverse events due to study drug, postoperative complications and mortality will be recorded before hospital discharge.

10.Pain evaluation

The intensity of postoperative pain both at rest and with coughing will be evaluated using the numeric rating scale (NRS, a 11-point scale where 0 indicates no pain and 10 indicates the worst pain). NRS score will be evaluated every 15min in the post-anesthesia care unit. For patients with a NRS pain score > 4, PCIA bolus will be firstly administered; in case of no significant improvement after three consecutive boluses, extra morphine 2-4 mg per time (maximum 0.1 mg/kg per hour) will be administered and repeated. NRS pain score (at rest and with movement) will be assessed at 4, 12, 24, 48, and 72 hours after surgery.

11.Rescue regiments for pain control

For patients in the ward with a NRS pain score > 4, PCIA bolus will be firstly administered; in case of no significant improvement after three consecutive boluses, extra morphine 2-4 mg per time will be administered and repeated.

12.Adverse events

1. Vital signs including heart rate, blood pressure and electrocardiogram will be monitored from end of anesthesia until 72 hours after surgery.
2. Investigators will review the data of vital sign monitor every 12 hours. Any abnormal vital signs will be recorded including the type, time of onset, duration and treatment.
3. The physicians and the nurses in the ward will be informed about the potential side effects of the drug and treatment plan. When side effects occur, it must be treated by the physician in the ward or the investigator. Any treatments will be recorded in the document.
4. Bradycardia is defined as heart rate < 50 bpm or a 30% decrease from baseline. If bradycardia occur, consider administering atropine 0.2-0.4mg mg IV. This may be repeated 5 to 10 minutes up later. If three attempts fail, physician can stop the drug and inform the investigator.
5. Tachycardia was defined as heart rate > 120 beats per minute (bpm) or a 30% increase from baseline. If tachycardia occur, fluid replacement with 300-500ml crystal will be given in the condition of hypovolemia. Otherwise, esmolol 20-50mg will be injected and repeated every 10 minutes. If three attempts fail, physician can stop the drug.
6. Hypotension is defined as systolic blood pressure < 90 mmHg or 20% decrease from baseline. If hypertension occur, fluid replacement with 250ml crystal will be given in the condition of hypovolemia and repeat if needed. Consider administering ephedrine 6mg i.v. (this may be repeated 5 to 10 minutes up later.) or continuous infusing dopamine. If all the treatments above failed, physicians can stop the drug and inform the investigator.
7. Hypertension is defined as systolic blood pressure > 180 mmHg or diastolic pressure <100mmHg or a 20% increase from baseline. If hypertension occur, consider administration of urapidil hydrochloride 5-10mg or nicardipine 0.2-0.5mg i.v. (this may be repeated 5 to 10 minutes up later.) or continuous infusing urapidil hydrochloride or nicardipine. If all the treatments above fail, physicians can stop the drug and inform the investigator.
8. If SpO_2_ decreases (<92% or 10% from baseline), considering application of nasal prongs for oxygen therapy(5L/min) and encouraging patient to have deep breathing/controlled coughing. If all the treatments above fail, mask oxygen-inspiration can be administered and physician can stop the drug and inform the investigator.
9. Over sedation is defined as Richmond Agitation Sedation Scale (RASS) ≤ -2. Respiratory depression is defined as respiratory rate < 8 bpm. If over sedation or respiratory depression occur, physician can stop the drug and inform the investigator.
10. If any other adverse event due to the drug occur, physician can stop the drug and inform the investigator.
11. If the drug is stopped, the investigator will provide another patient-controlled pump without dexmedetomidine.
12. If nausea, retching or vomiting occur, consider administering ondansetron 5mg IV.

13. All serious adverse events will be reported to the investigator and the local ethics committee as soon as possible, but no later than 24 hours from the time the study team received knowledge of the event.

**Statistical analysis**

1. Sample size calculation

In a previous study, a 24-hour infusion of dexmedetomidine (at a rate of 0.08 μg/kg/h) decreased morphine consumption by 30%.^1^ Considering that our patients are old and PCIA will be used for 3 days, the dexmedetomidine concentration in the PCIA formula is decreased and, therefore, the background infusion rate is lower (a background infusion rate of 2μg/h, that is about 0.02 to 0.04μg/kg/h depending on patient’s body weight from 50 to 90 kg) .We assumed that the addition of dexmedetomidine will reduce morphine consumption by 20%, i.e., from 50 mg in the CTRL group to 40 mg in the DEX group. With the significance 0.05 and power 0.8, 24 patients in each arm are required. Taking a dropout rate of about 20%, we planned to enroll 29 patients in each arm.

1. Outcome analysis
2. Primary analysis. Cumulative morphine consumption will be compared with independent sample t-test.
3. Secondary analyses. NRS pain score will be compared with Mann-Whitney U-test.
4. Incidence of postoperative complications, study related adverse events and mortality will be compared with Chi-square test or Fisher’s exact test.
5. Missing data. Replace the missing data with a worst value defined by the study protocol.
