# Supplementary material for: Dexmedetomidine in combination with morphine improves postoperative analgesia and sleep quality in elderly patients after open abdominal surgery: A pilot randomized control trial
Source: PLoS One. 2018 Aug 14;13(8):e0202008. doi: 10.1371/journal.pone.0202008 (PMC6091958; doi:10.1371/journal.pone.0202008)
Supplement: S4 File — (PDF) [file pone.0202008.s004.pdf]

北京大学第一医院临床试验伦理委员会伦理审查批件

伦理审查编号：(2014）药物非注册第（24）号

项目名称	右美托咪定用于老年患者术后镇痛的有效性研究		
CFDA 批件号	/		
申办者	北京大学第一医院　麻醉科		
临床研究科室	麻醉科	主要研究者	王东信
送审文件	具体见随附“北京大学第一医院临床试验伦理委员会审查文件清单”		

本伦理委员会的人员组成和工作程序符合中国 GCP 以及国家相关规定

伦理审查方式：■会议审查　■快速审查

会议审查时间：2014 年 09 月 10 日	会议地点：北京大学第一医院伦理委员会会议室
审查委员	见随附“伦理委员会会议签到表”。
审查意见	1. 经本伦理委员会审查：同意进行该临床研究。<br>意见和建议：无。<br>2. 该研究进行过程中将接受伦理委员会的定期跟踪审查？■是　□否<br>定期跟踪审查频率：□ 6 个月　■12 个月

注意事项：

1. 本项临床试验应当在伦理委员会同意进行之日起 1 年内实施。逾期未实施的，本审查批件自行废止。
2. 研究应遵循本伦理委员会批准的方案执行，须符合 CFDA/GCP 和《赫尔辛基宣言》的原则。
3. 自同意研究之日起，每隔 12 个月伦理委员会的定期跟踪审查（审查频度可能根据实际进展情况改变）；请在定期跟踪审查到期前 1 个月递交《定期跟踪审查表》。
4. 研究过程中，对研究方案和知情同意书等相关文件所作的任何修改，请交《修正案申请表》及“送审文件清单”中规定相关资料，并得到伦理委员会审查同意该修正后方可实施。
5. 发生严重不良事件或影响研究风险受益比的非预期不良事件，在向 CFDA 上报的同时向伦理委员会作书面通报，可以使用 CFDA 的《严重不良事件报告表》或本伦理委员会公布的《严重不良事件/非预期不良事件报告表》或其他有相关内容的报告表，但外文的报告需要有中文摘要。伦理委员会有权根据对其评估做出新的决定。
6. 不依从或违反方案应及时提交《不依从或违反方案报告表》。
7. 提前终止研究应及时提交《研究方案提前终止报告表》。
8. 研究完成后提交《研究结题报告表》和临床试验总结报告。
9. 及时书面报告其他伦理委员会的重要决定。

主任委员或副主任委员签字：[illegible]

北京大学第一医院临床试验伦理委员会（盖章）

2014 年 11 月 6 日

伦理委员会地址：北京市西城区大红罗厂街 6 号　邮编：100034　联系电话：010-66119025
