# Supplementary material for: Dexmedetomidine in combination with morphine improves postoperative analgesia and sleep quality in elderly patients after open abdominal surgery: A pilot randomized control trial
Source: PLoS One. 2018 Aug 14;13(8):e0202008. doi: 10.1371/journal.pone.0202008 (PMC6091958; doi:10.1371/journal.pone.0202008)
Supplement: S5 File — (DOCX) [file pone.0202008.s005.docx]

**Clinical Research Ethics Committee of Peking University First Hospital**

**Approval File**

**Project No.:( 2014) Drug test off-lable No.[24]**

| **Title:** The effect of dexmedetomidine in combination with morphine on postoperative analgesia and sleep quality in elderly patients after open abdominal surgery: a pilot randomized control trial | | |
| --- | --- | --- |
| **CFDA Approval No.:** / | | |
| **Applicant:** Department of Anesthesiology and Critical Care | | |
| **Department:** Anesthesiology and Critical Care | | **PI:** Wang Dongxin |
| **Review files:** see attachments (Review checklist of clinical research ethics committee of Peking University First Hospital) | | |
| **The composition of the ethics committee and working procedure are in accordance with GCP and other related regulations and laws in China.** | | |
| **Review type: ■**Meeting review, **■**Quick review | | |
| **Meeting Review date:2014-9-10** | **Meeting place:** Conference room of ethics committee of Peking University First Hospital | |
| **Review committee:** see attachment | | |
| **Result of review:**  **1.** According to Helsinki declaration, GCP and other related regulations and laws in China, the Committee approved this study protocol.  **2.** Is there any tracking review during the study period? **Yes.**  Tracking review time: every 12 months | | |
| **Notice:**  1.The clinical trial must be administrated within 1 year from the issued date of the ethics committee approved.  2.Clinical trial must be administrated according to the approved protocols and according to Helsinki declaration and GCP/CFDA.  3.Tracking review will be administrated 12 month later from the issued date (The frequency of the tracking review will change according to the study). Please submit a tracking review file 1 month before the valid date.  4.Any change in study protocol and consent inform must be submitted to the committee for approval. without approval of the committee, the new protocol cannot be administrated.  5.If any severe adverse events or unanticipated adverse events happened, please submit a report to the ethics committee. The ethics committee would make new decision on this review.  6.Please submit non-compliance report or protocol violation report in time if necessary.  7.If the study is preliminarily stopped, please submit a report  8.If the study is completed, please submit a report  9.Please submit any other written report to the ethics committee if necessary. | | |
| Director of committee: Guo Xiaohui  Clinical Research Ethics Committee of Peking University First Hospital  Issued date 11/6/2014 | | |
| Address: Dahongluochang Street No. 6, Beijing China.  TEL: +86 010 66119025.  Code:100034 | | |
